# The modified woodward procedure via a limited incision for Sprengel deformity: a retrospective study on efficacy and cosmesis

**DOI:** 10.3389/fped.2026.1760390

**Published:** 2026-05-28

**Authors:** Peng Zhang, Lianbin Su, Zijie Sun, Jun Xiao, Hengyan Zhuge, Kefeng Xu, Gengyang Jin, Qi Liu

**Affiliations:** 1 Department of Orthopedics, The 904th Hospital of Joint Logistic Support Force of PLA, Wuxi, China; 2 Department of Pediatric Orthopedics, The First Affiliated Hospital of Xiamen University, Xiamen, China

**Keywords:** cosmetic outcome, limited incision, minimally invasive surgery, modified woodward procedure, shoulder function, Sprengel deformity

## Abstract

**Background:**

The Woodward procedure is a standard surgical treatment for Sprengel deformity (SD), but its classic execution requires extensive tissue dissection. This study aimed to evaluate the outcomes of a Modified Woodward Procedure via a Limited Incision (MWP-LI), designed to minimize tissue trauma while enhancing functional and cosmetic results.

**Methods:**

We retrospectively reviewed eight consecutive SD patients (5 Cavendish grade III, 3 grade II) treated with the MWP-LI between January 2022 and December 2023. The key modifications included a short midline incision, creation of a limited trapezius-rhomboid musculofascial flap, dynamic integrated release under continuous scapular traction, and selective scapulothoracic release. Tension-reducing cosmetic closure was employed.

**Results:**

At a mean follow-up of 26.5 months, the mean active shoulder abduction improved significantly by 58.0° (from 96.5° ± 11.8° to 154.5° ± 10.6°, *p* < 0.001). The mean Cavendish grade improved by 1.8 grades. We recognize that this follow-up duration is insufficient to assess long-term growth-related recurrence; ongoing follow-up is planned. All parents reported high satisfaction with the outcomes. No instances of scapular winging, neurovascular injury, or other major complications were observed.

**Conclusion:**

The MWP-LI offers a reliable, less invasive, and cosmetically superior alternative for correcting SD.

**Level of evidence:**

Therapeutic Level IV.

## Introduction

Sprengel deformity (SD) is the most common congenital anomaly of the shoulder girdle, resulting from a failure of caudal scapular migration during embryonic development (1, 2). This condition presents with both cosmetic deformity and functional limitations in shoulder abduction and flexion (2, 4). Surgical intervention is typically indicated for moderate to severe cases (Cavendish grades II-IV) to address these concerns (5, 6).

Among the various surgical techniques described (3, 7–11), the Woodward procedure has been established as a cornerstone for the correction of SD (5, 12). However, the classic Woodward technique involves an extensive midline incision and detachment of the trapezius and rhomboid muscles from the occiput to the lower thoracic spine. This extensive dissection is associated with significant tissue trauma, prominent scarring, increased postoperative pain, and prolonged recovery (2, 5).

In recent years, efforts to reduce surgical invasiveness have led to the development of endoscopic-assisted techniques (13, 14). While these approaches minimize superficial dissection, they present a steep learning curve and may compromise the anatomic reattachment of the trapezius and rhomboid muscles, raising concerns about postoperative scapular winging and long-term stability (2, 5).

To bridge this gap, we developed a refined surgical approach: the modified Woodward procedure via a limited incision (MWP-LI). This technique strategically balances minimal access surgery with the core principles of the open Woodward procedure. Its key modifications include: a short midline incision, a limited trapezius-rhomboid musculofascial flap, and a dynamic integrated release under continuous scapular traction. This study retrospectively evaluates the safety, efficacy, and cosmetic outcomes of the MWP-LI in eight consecutive SD patients.

## Materials and methods

### Patient demographics and study design

This single-center, retrospective cohort study was approved by the Institutional Review Board of The First Affiliated Hospital of Xiamen University. Written informed consent was obtained from the legal guardians of all participants prior to surgery. We reviewed the records of all consecutive patients diagnosed with SD who underwent the MWP-LI between January 2022 and December 2023.

The inclusion criteria were: (1) a diagnosis of SD with Cavendish grade II or higher; (2) primary surgical correction performed using the MWP-LI technique as described below; and (3) a minimum clinical and radiographic follow-up of 24 months. Patients with prior surgery on the affected shoulder girdle were excluded. A total of eight patients met these criteria. Preoperative demographics and clinical characteristics are summarized in Table 1.

**Table 1:** Preoperative patient demographics and clinical characteristics (*n* = 8).

Characteristic	Value
Sex (Male:Female)	2:6
Mean Age at Surgery (years)	4.5 (range, 3–6)
Affected Side (Left:Right:Bilateral)	7:1:0
Preoperative Cavendish Grade	
Grade II	3
Grade III	5
Mean Preoperative Abduction (°)	96.5 ± 11.8
Associated Anomalies (n, %)	4 (50%)
Klippel-Feil Syndrome	2
Scoliosis	1
Omovertebral Bone	3

### Surgical technique: the MWP-LI

All procedures were performed using the MWP-LI by the senior author under general anesthesia with intraoperative neuromonitoring. Patients were positioned prone with the affected shoulder elevated.

A midline dorsal incision was made, extending from the level of the superior medial angle (typically at T1–T2) of the contralateral scapula to 1.5–2 cm above the level of its inferior angle (typically at T6–T7)—approximately half the length of the traditional Woodward incision.

The trapezius and rhomboid muscles were exposed. In contrast to the conventional extensive detachment, our approach involved a strategic limited release. The spinal attachments were divided only from the level of the superior medial angle of the contralateral scapula to the level of its inferior angle, in order to create a trapezius-rhomboid musculofascial flap.

A critical technical aspect involved synchronized scapular manipulation and deep dissection under dynamic tension. While the assistant applied downward, inward and external rotation pressure on the scapula, in conjunction with simultaneous arm abduction, the following procedures were performed:

1. The tense, fibrotic levator scapulae was identified and divided.
2. Any omovertebral bone or fibrous connections were meticulously excised.
3. The prominent superomedial scapular angle was exposed subperiosteally and resected.

With continued dynamic manipulation, shoulder abduction was assessed. When subscapular tissue tension limited full abduction, adhesions between the scapula and thoracic wall were selectively released by blunt digital dissection until near-complete passive abduction (≈180°) was achieved.

**Figure 1:**
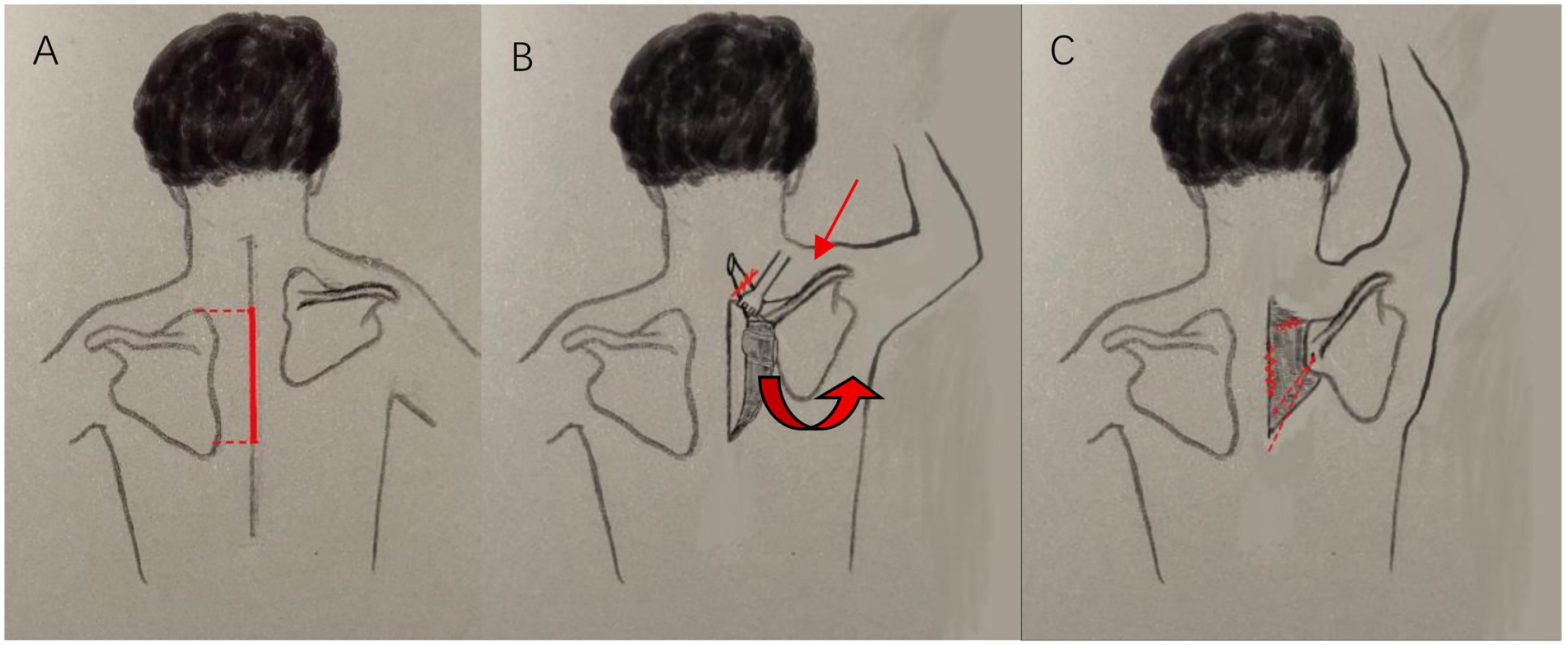
Schematic illustration of the MWP-LI. **(A)** Comparison between the traditional Woodward incision (black line) and the modified limited incision (red line). **(B)** After the preparation of the limited musculofascial flap, release of restrictive structures (e.g., levator scapulae, omovertebral bone) is performed under continuous traction involving downward, inward and external rotation (as indicated by the red arrows), which was applied by an assistant. **(C)** The scapula is fixed in its corrected caudal position with non-absorbable sutures (red dashed line). The musculofascial flap is subsequently re-sutured to the spinal processes.

With the scapula held in the corrected position, a 2.0 mm hole was then drilled 1 cm lateral to the medial scapular spine border, thus providing a solid bony anchor point. Two #2 non-absorbable sutures were then passed through the hole and anchored to the two spinous processes at the level corresponding to the inferior angle of the contralateral scapula, thus maintaining a 30–45° angle with the spine. The trapezius-rhomboid musculofascial flap was then reattached to the spinous processes at their new caudal positions, approximately one vertebral level downward to maintain the scapula in its corrected position and prevent recurrence. Hemostasis was achieved, and the wound was closed in layers using tension-reducing cosmetic suturing techniques without drainage. Figure 1 provides a schematic overview of the key steps of the MWP-LI

### Postoperative management

**Figure 2:**
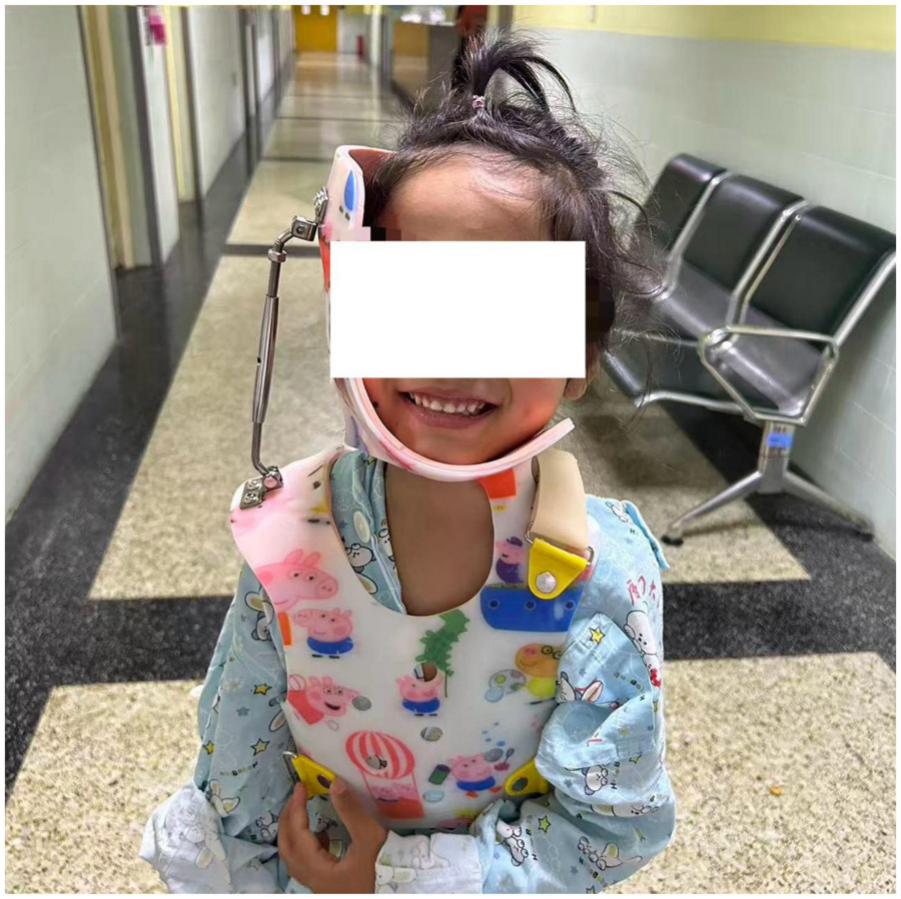
Postoperative custom-made shoulder brace. The brace uses an adjustable-length support rod to apply downward pressure on the affected scapula, counteracting pain-induced shoulder shrugging. It does not restrict glenohumeral motion.

Immediately after surgery, a custom-made shoulder brace was applied. The brace was worn continuously for 4 weeks, except during supervised active-assisted range-of-motion exercises. The primary purpose of the brace is to antagonize the patient's pain-induced compensatory shoulder shrugging, thereby maintaining the distance between the cervical spine and the affected scapula, rather than providing rigid immobilization of the shoulder joint. As shown in Figure 2 (a patient with right-side SD wearing the brace postoperatively), the brace applies downward pressure on the affected scapula via an adjustable-length support rod connecting the cervico-occipital portion and the shoulder unit of the custom orthosis. It does not restrict shoulder abduction or external rotation; no specific abduction or external rotation angle is preset in the orthosis.

Beginning on postoperative day 2, daily supervised active-assisted elevation exercises were initiated. In most patients, passive elevation to nearly 180∘180∘ (arm touching the ear) was achievable within the first week. The brace was removed for each training session and reapplied immediately afterward.

Full, unrestricted activities were permitted after 4 weeks. In our experience, if patients do not achieve nearly full active abduction within 1 month postoperatively, they rarely do so later; thus, early intensive training is critical.

### Outcome assessment

Patients were evaluated preoperatively and at the latest follow-up. Functional outcome was assessed by measuring active shoulder abduction. Cosmetic outcome was graded by two independent surgeons using the Cavendish classification; any discrepancy was resolved by consensus. Parental satisfaction was assessed via a non-validated but structured questionnaire covering satisfaction with shoulder function, cosmetic appearance, and the surgical scar (rated on a 5-point Likert scale from “Very Dissatisfied” to “Very Satisfied”). All complications were recorded.

### Statistical analysis

Data were analyzed using SPSS Statistics version 26 (IBM Corp.). Preoperative and postoperative abduction angles were compared using a paired-sample *t*-test, with *p* < 0.05 considered statistically significant.

## Results

All eight patients successfully underwent the MWP-LI without conversion to a standard, more extensive open approach. The mean follow-up duration was 26.5 months (range: 24–36 months).

**Figure 3:**
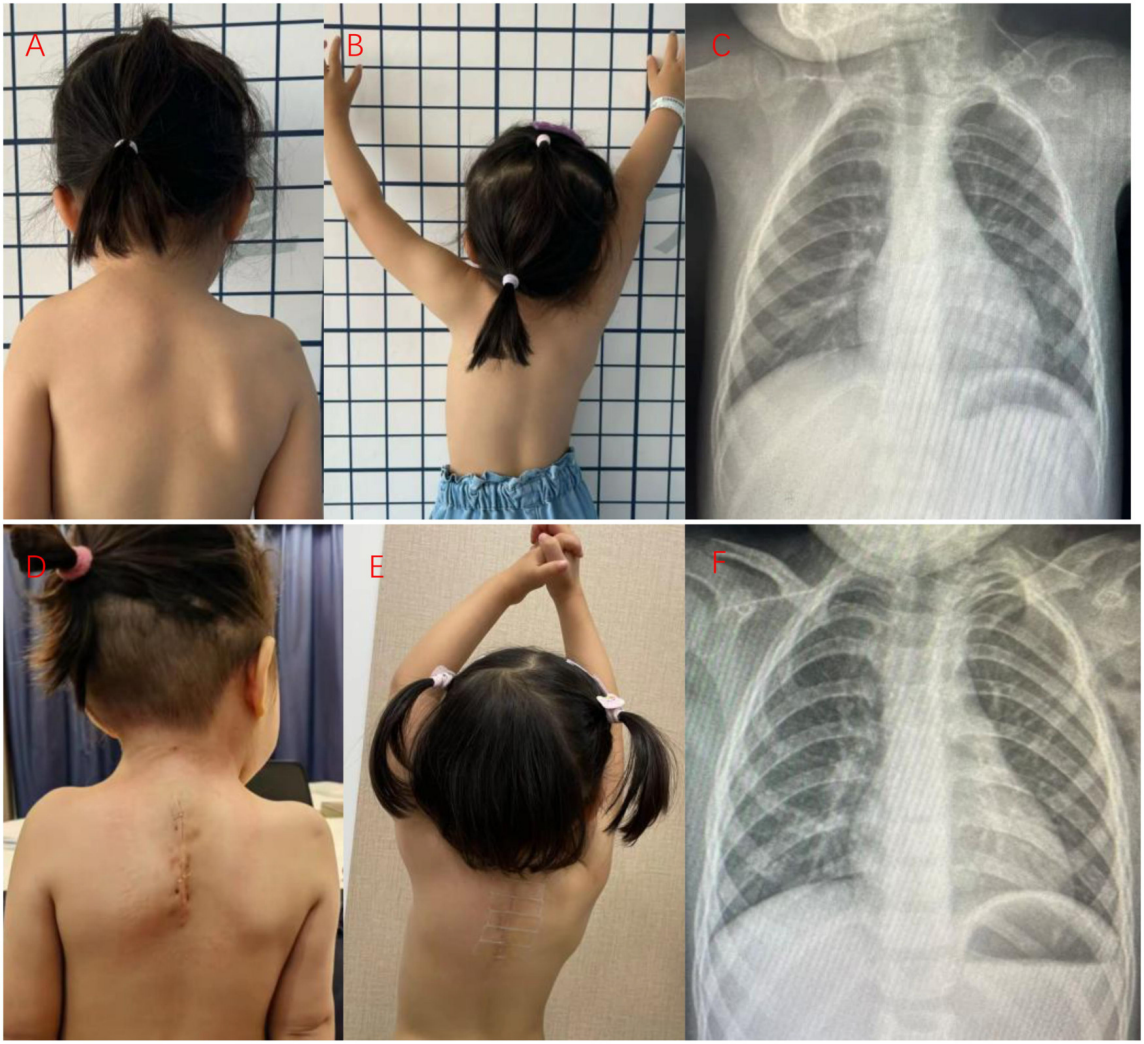
Pre- and postoperative comparison of a typical case associated with klippel-feil syndrome (3-year-old female, cavendish grade III). **(A)** Preoperative posterior view clearly demonstrating elevated scapulae and short neck deformity. **(B)** Preoperative maximum active shoulder abduction photograph showing functional limitation. **(C)** Preoperative anteroposterior (AP) radiograph revealing high scapulae. **(D)** Postoperative dorsal view showing markedly improved scapular position with bilateral symmetry and an aesthetically pleasing short scar. **(E)** Postoperative maximum active shoulder abduction demonstrating near-normal range of motion. **(F)** Postoperative AP radiograph confirming scapular descent to the normal level.

**Functional Outcomes:** A significant improvement in mean active shoulder abduction was observed, from 96.5° ± 11.8° preoperatively to 154.5° ± 10.6° postoperatively (*p* < 0.001), representing a mean gain of 58.0° (Tables 2, 3). This improvement enabled all patients to perform age-appropriate overhead activities.
**Cosmetic Outcomes:** Cosmetic appearance improved markedly. The mean Cavendish grade improved from 2.6 preoperatively to 0.8 postoperatively, an average improvement of 1.8 grades (Table 2). Postoperatively, most patients (7/8, 87.5%) were rated as Cavendish grade 0 or I. A representative case is shown in Figure 3.

**Table 2:** Preoperative and postoperative functional and cosmetic outcomes (*n* = 8).

Outcome Measure	Preoperative	Postoperative	*P*-value
Shoulder Abduction (°)			
Mean ± SD	96.5 ± 11.8	154.5 ± 10.6	< 0.001
Range	(80–115)	(140–170)	
Cavendish Grade (*n*)			
Grade 0	0	3	
Grade I	0	4	
Grade II	3	1	
Grade III	5	0	

**Table 3:** Individual patient demographic, clinical, and outcome data (*n* = 8).

Patient	Age (yr)/Sex/Side	Preop Cavendish	Postop Cavendish	Preop ABD (°)	Postop ABD (°)	Associated anomalies	Follow-up (months)	Complications
1	3/F/L	III	I	115	150	None	36	None
2	4/F/R	II	0	89	163	Klippel-Feil	25	None
3	5/M/L	III	I	84	145	Omovertebral bone	28	Superficial erythema
4	3.5/F/L	II	0	107	165	None	26	None
5	6/F/L	III	II	80	140	Scoliosis, Omovertebral bone	25	None
6	3.5/F/L	III	I	102	170	None	24	None
7	5/F/L	II	0	98	155	Klippel-Feil, Omovertebral bone	24	None
8	6/M/L	III	I	97	148	None	24	None

ABD, active shoulder abduction; F, female; M, male; L, left; R, right; wks, weeks.

### Intraoperative and postoperative course

Intraoperative neuromonitoring (IONM), including continuous somatosensory evoked potentials (SEP) and motor evoked potentials (MEP), was employed in all cases. Throughout the critical surgical stages—specifically during scapular release, omovertebral bone resection, and caudal displacement of the scapula—no significant neurophysiological alerts were observed. An alert was predefined as a decrease in MEP amplitude of >50% or a prolongation of SEP latency of >10% from baseline. No major perioperative complications occurred. Specifically, there were no instances of brachial plexus palsy, vascular injury, deep infection, or clinically evident scapular winging. One patient developed superficial wound erythema that resolved promptly with a short course of oral antibiotics.

### Parental satisfaction

All eight parents or guardians completed the satisfaction survey. Seven (87.5%) reported being “Very Satisfied” and one (12.5%) reported being “Satisfied” with both the functional improvement and the cosmetic outcome. The minimal and well-concealed scarring was specifically commended in the feedback.

## Discussion

The principal finding of this study is that the MWP-LI achieved a mean functional improvement of 58.0° in shoulder abduction, which appears notably higher than the 40–50° range reported in most previous series (15). We attribute this directly to our intraoperative goal: achieving passive, tension-free shoulder abduction with the arm touching the ear (≈180°) in every patient.

Several technical features enable this endpoint without increasing risk:

First, complete and customized release—Based on the senior author's experience with nearly 20 traditional open Woodward procedures prior to developing this technique, we perform a meticulous release of all restrictive structures, including scapulothoracic adhesions via blunt digital dissection, which is often underemphasized. The limited incision does not compromise the completeness of release because we know precisely where to dissect.

Second, superior fixation strategy—Instead of simply reattaching the trapezius-rhomboid flap (which may undergo elastic recoil), we anchor the scapula using a non-absorbable suture passed through a bone tunnel at the medial border of the scapular spine. This location maintains both caudal descent and adequate external rotation while still allowing physiological mobility (not rigid fixation), preventing recurrence without restricting range.

Third, minimizing collateral damage—By limiting detachment to only the necessary structures, we reduce postoperative pain and muscle weakness, enabling early, nearly pain-free active-assisted training within the first postoperative week. In our experience, if patients do not achieve nearly full active abduction within 1 month postoperatively, they rarely do so later.

Fourth, our cohort consisted of Cavendish grades II-III (no grade IV), young children (3–6 years) with good tissue elasticity.

Considerations for Cavendish grade IV deformity—No grade IV patient was included in this series. The MWP-LI via a limited incision is a relative contraindication for severe grade IV deformity, particularly when a thick, ossified omovertebral bone is fused to the cervical spine or occiput. For such cases, we recommend either a traditional long-incision Woodward procedure or conversion to an extended incision if MWP-LI is attempted. Therefore, MWP-LI should be applied cautiously in grade IV deformity, ideally only by surgeons with extensive prior open experience (e.g., >20 cases) and intraoperative neuromonitoring.

Clavicular osteotomy was not performed in any patient in this series. We reserve clavicular osteotomy for specific scenarios: adolescent/adult SD, concomitant clavicular malformation, or intraoperative neuromonitoring evidence of excessive brachial plexus tension after scapular descent.

The favorable outcomes of the MWP-LI are attributed to specific technical modifications that enhance both the efficacy and safety profile of the correction. First, the dynamic integrated release under continuous, controlled traction facilitates a more complete and incremental mobilization of the scapula. This titrated approach allows for thorough release of restrictive structures while adhering to the critical principle of avoiding excessive correction, which is recognized as a key measure in preventing brachial plexus injury (6). Second, and central to this technique, is the achievement of this correction through a strategically limited surgical exposure. The incision length is reduced by approximately 50%, and the creation of the trapezius-rhomboid musculofascial flap is precisely confined. This represents a significant departure from the extensive dissections of classic approaches. Crucially, this limited access does not compromise the fundamental tenet of the Woodward procedure: it still permits direct visualization and anatomical reattachment of the musculofascial flap, ensuring reliable soft tissue reconstruction for long-term scapular stability.

When compared to other contemporary surgical strategies, the technical nuances of the MWP-LI confer distinct advantages:

**Compared to the classic Woodward procedure**, the MWP-LI offers a demonstrable reduction in tissue trauma, directly translating to a smaller, more cosmetic scar and potentially less postoperative morbidity, while preserving the capacity for robust anatomical repair.
**Compared to other modified open techniques, such as the anchoring suture modification by Alsiddiky et al.** (5), the MWP-LI introduces refinements in fixation strategy and exposure. Alsiddiky et al. conscientiously avoided bringing the inferior scapular angle down to the contralateral level to prevent brachial plexus traction, opting to anchor the scapula to the lower thoracic spine (T11/T12) (5). This necessitates a more extensive caudal dissection. In contrast, the MWP-LI secures the scapula at a more cephalad level (corresponding to T6–T7). Furthermore, our technique employs a bone tunnel through the robust scapular spine, providing a solid bony anchor point. This contrasts with the suture placement “1 cm lateral to the vertebral edge” in their technique (5), a location where bone may be thinner and potentially less reliable for long-term fixation, which might influence the risk of recurrence. Importantly, the MWP-LI achieves this secure, superior fixation through its limited incision, suggesting a more efficient and potentially less traumatic route to a stable correction.
**Compared to the endoscopic-assisted Woodward technique**, the MWP-LI provides a critical advantage in ensuring musculoskeletal balance. Endoscopic approaches, while minimizing skin incisions, omit the reattachment of the trapezius and rhomboid muscle due to technical constraints (13, 14). An inadequate or imbalanced scar repair could potentially lead to altered scapular kinematics and affect long-term shoulder function. The MWP-LI, utilizing a limited open exposure, guarantees a direct and robust re-fixation of these key stabilizers under visual guidance, thereby maintaining proper muscular force couples.

In summary, the success of MWP-LI does not rely on the small incision *per se*. The true essence of the Woodward procedure—complete, customized release, physiological anchoring that allows mobility, and early pain-free active training—is preserved and even enhanced. The limited incision merely reduces collateral damage, enabling faster recovery. The most common cause of recurrence is not the surgical approach but fixating the scapula in a new rigid position without restoring its natural freedom of motion. Our technique avoids this by using a non-rigid suture anchor at the scapular spine, which maintains both descent and external rotation while permitting dynamic scapular movement.

### Limitations

We acknowledge several limitations inherent to this study. Its retrospective design and the lack of a concurrent control group preclude definitive comparative conclusions regarding superiority. The sample size, though substantial for this rare condition, remains small. Furthermore, the mean follow-up of 26.5 months (maximum 36 months) is adequate for assessing early functional recovery, complication rates, and short-term recurrence, but is insufficient to evaluate long-term outcomes such as growth-related recurrence. Given that scapular growth continues until approximately 10–12 years of age, and literature-reported recurrences often occur after 5 years or more, we are continuing follow-up of this cohort with planned assessments at 5 and 10 years postoperatively. Readers should interpret our results as indicative of short-to-mid-term outcomes only. Future prospective studies with larger cohorts, direct comparative groups, and extended follow-up are warranted to validate these promising preliminary findings.

## Conclusion

In conclusion, the Modified Woodward Procedure via a Limited Incision (MWP-LI) represents a clinically effective and refined approach for the surgical correction of SD. Our study demonstrated that this technique yields substantial functional improvement, with a mean gain of 58.0° in shoulder abduction, and excellent cosmetic outcomes, alongside a favorable safety profile. The MWP-LI achieves these results through key technical refinements—including a significantly shortened incision, a strategically limited musculofascial flap, and dynamic integrated release—which collectively reduce tissue trauma without compromising the principles of anatomical reconstruction and stable fixation. By offering a balance between minimally invasive access and reliable open repair, the MWP-LI presents a valuable alternative to both the classic Woodward procedure and emerging endoscopic-assisted techniques. It addresses important concerns related to cosmesis, surgical morbidity, and musculoskeletal balance, which are central to patient and parental satisfaction. While the present findings are promising, further prospective and comparative studies with longer follow-up are encouraged to confirm the long-term durability and broader applicability of this technique.

## Data Availability

The original contributions presented in the study are included in the article/Supplementary Material, further inquiries can be directed to the corresponding author.
